# Global effect of copper excess and deficiency in *Saccharomyces cerevisiae* proficient or deficient in nonsense-mediated mRNA decay

**DOI:** 10.1016/j.ygeno.2025.111020

**Published:** 2025-02-22

**Authors:** Xinyi Zhang, Sunday Olaniyan, Xiayan Li, Bernd Zechmann, Mary Lauren Benton, Bessie Kebaara

**Affiliations:** a Department of Biology, Baylor University, Waco, TX, USA; b Department of Computational Medicine and Bioinformatics, University of Michigan, Ann Arbor, MI, USA; c Center for Microscopy and Imaging, Baylor University, Waco, TX, USA; d Department of Computer Science, Baylor University, Waco, TX, USA

**Keywords:** mRNA decay, Nonsense-mediated mRNA decay, *Saccharomyces cerevisiae*, Copper homeostasis, Bio-metals

## Abstract

The highly conserved nonsense-mediated mRNA decay (NMD) pathway was initially identified as an mRNA surveillance pathway. NMD is now also known to have multiple functions including precise regulation of gene expression. In *Saccharomyces cerevisiae*, about 5–10 % of the transcriptome is regulated by the NMD pathway. Previous studies found environmental condition-specific regulation of transcripts by NMD in *S. cerevisiae*. In this study, we examined the effect varying copper levels have on global regulation of mRNAs by NMD. Specifically, the consequences of copper excess and deficiency on cellular ultrastructure and transcriptomes of *S. cerevisiae* cells with a functional and non-functional NMD pathway was investigated. Copper excess or deficiency resulted in enlarged vacuoles in yeast cells relative to cells grown in normal growth conditions. Additionally, yeast cells with a functional NMD pathway had dilated endoplasmic reticulum (ER) when exposed to elevated copper levels. In elevated copper levels dilated ER were not observed in cells with a non-functional NMD pathway. Furthermore, copper deficiency led to widespread changes in gene expression relative to the normal growth and elevated copper conditions. Significant enrichments for Molecular function (MF) included transmembrane transporter activity and helicase activity for transcripts upregulated in complete minimal (CM) only. For transcripts upregulated in both CM and 100 μM copper, significant enrichments for MF were found in structural constituent of cell wall, ferric-chelate reductase (NADPH) activity, metal ion and DNA binding. Transcripts upregulated specifically in low copper were greatly enriched for categories related to RNA binding and RNA metabolic processes.

## 1. Introduction

The nonsense-mediated mRNA decay (NMD) pathway is highly conserved in eukaryotes. NMD was initially discovered as an mRNA surveillance system that degrades transcripts bearing premature termination codons (PTCs), thus preventing the synthesis of potentially harmful truncated proteins [1,2]. In addition to mRNA surveillance, the NMD pathway functions in protection from viral pathogens and precise regulation of gene expression through degradation of protein coding natural mRNAs [3–5]. In *Saccharomyces cerevisiae*, NMD factors are not essential for growth under normal growth conditions, while knockout of NMD factors in mammalian cells, causes embryonic lethality [6]. Therefore, *S. cerevisiae* has become the ideal model to study the regulation of natural mRNAs by the NMD pathway. Approximately 5–10 % of the yeast transcriptome is affected when NMD is non-functional [7,8].

Natural mRNAs that are direct NMD targets are degraded by the pathway because they contain NMD inducing features. The known NMD targeting features of natural mRNAs in yeast include, mRNAs with atypical long 3′-untranslated regions (UTR), mRNAs with a suboptimal start codon context that may be subject to out-of-frame translation initiation at a different AUG codon within the open reading frame (ORF), some mRNAs that contain translated upstream open reading frames (uORFs), mRNAs that are subject to frameshifting, inefficiently spliced pre-mRNAs that enter the cytoplasm and certain alternative splicing events [7,9–14]. From yeast to humans, activation of NMD requires the function of three principal up-frameshift (UPF) factors: Upf1, Upf2 and Upf3 [2,15,16]. These three proteins interact with each other, the ribosome, and multiple translation and mRNA decay factors [17]. *UPF1* is the central NMD regulator, with *UPF2* and *UPF3* regulating *UPF1* function.

NMD plays an important role in bio-metal homeostatic mechanisms, including magnesium, copper, zinc, and iron homeostasis [18]. Copper is an essential metal to eukaryotes, and serves as a co-factor for enzymes, including cytochrome *c* oxidase required for respiration, Cu, Zn-superoxide dismutase (SOD1) for oxidative stress protection and multicopper oxidases, *FET3* and *FET5* in *S. cerevisiae* [19,20]. Copper-responsive transcriptional regulation is of vital importance in fungi, where a variety of factors control genes required for copper acquisition and detoxification [21]. Copper toxicity can result in generation of reactive oxygen species (ROS). Build-up of ROS can damage lipids, proteins, and nucleic acids. Thus, organisms have evolved sophisticated homeostatic mechanisms for copper uptake, transport, and utilization since copper is essential but toxic in excess. Extensive evolutionary conservation is observed in copper homeostatic mechanisms between human and *S. cerevisiae*, making *S. cerevisiae* a suitable model organism for investigating molecular mechanisms of copper homeostasis [22,23].

A Prior global gene expression profiling study found that 128 genes had significant changes in mRNA expression levels in response to changes in copper levels [24]. An additional study on differential gene expression under copper excess and deficiency found a limited set of genes responded to copper levels [25]. Furthermore, global expression profiling studies have found that genes involved in copper homeostasis in *S. cerevisiae* are regulated in an NMD-dependent manner [8,26]. However, the extent to which changes in copper levels lead to wide-spread changes in the transcriptome and cellular ultrastructure of wild type and NMD mutant yeast strains is undetermined.

Changes in environmental conditions causes differential regulation of mRNAs involved in copper homeostasis by NMD. These mRNAs include *MAC1*, *PCA1*, *COX17* and *FRE2* [27–31]. To understand how copper globally affects the regulation of mRNAs by the NMD pathway, we first grew wild type and NMD mutant cells under varying copper levels and subsequently measured total cellular copper levels in identical growth conditions. NMD mutants accumulated significantly higher levels of copper relative to wild type in complete minimal (CM) conditions (normal growth). We further observed the ultrastructure of wild type and NMD mutant yeast cells exposed to varying copper levels and found enlarged vacuoles in low copper and high copper conditions. Notably, wild type cells displayed dilated Endoplasmic Reticulum (ER) in elevated copper levels but not in the NMD mutant. Transcriptome analysis of wild type and NMD mutant (*upf1Δ*) *S. cerevisiae* strains grown under varying copper levels revealed changes in the transcriptome; specifically low copper levels led to widespread changes in gene expression. Gene Ontology (GO) enrichment analysis on transcripts upregulated in NMD mutants relative to the wild type strain identified enrichment in biological processes (BP) and molecular function (MF). Significant enrichments for MF included transmembrane transporter activity and helicase activity for transcripts upregulated in CM only in the NMD mutant (*upf1Δ*). For transcripts upregulated in both CM and 100 μM copper, significant enrichments for MF were found in structural constituent of cell wall, ferric-chelate reductase (NADPH) activity, metal ion and DNA binding. Transcripts upregulated in low copper specific were greatly enriched for categories related to RNA binding and RNA metabolic processes.

## 2. Materials and methods

### 2.1. Yeast cell growth and RNA isolation

All *S. cerevisiae* strains used in this study are from the W303 genetic background [32,33]. The wild type and NMD mutant strains were grown in complete minimal (CM), low copper complete minimal, and complete minimal supplemented with 100 μM copper (CuSO_4_). To attain low copper conditions, the media contained yeast nitrogen base without copper and iron (YNB-CuSO_4_-FeCl_3_) and 100 μM of the extracellular copper chelator, Bathocuproinedisulfonic acid (BCS) (Sigma-Aldrich). Glassware used in these experiments was soaked in 10 % nitric acid overnight to remove trace amounts of copper and dried out. All yeast cells used for low copper conditions were initially grown to saturation in CM media then sub-cultured into copper deficient media in acid washed glassware. For growth under high copper conditions, as with low copper conditions, the yeast cells were first grown to saturation in CM media then sub-cultured into CM media supplemented with 100 μM copper (CuSO_4_). In each case, cells were grown to mid-log phase (OD_600_ of 0.4–0.6) and harvested by centrifugation (4000 *g*/5 mins). Cell pellets were frozen on dry ice and then stored at −80 °C until RNA isolation. Total RNA was extracted from harvested cells using hot phenol method.

### 2.2. Measurement of total copper levels

Yeast cells were grown in CM, low copper (100 μM BCS), 100 μM copper and 600 μM copper. 600 μM copper was included because we previously found that NMD mutants tolerate high levels of copper above 600 μM compared to wild type strains [34]. Copper content was measured in triplicate using inductively coupled plasma mass spectrometry (ICP-MS) from 1 ml of yeast cells grown to an OD_600_ of 1.0 (1 × 10^7^ cells/ml). Cells were harvested by centrifugation for 2 min, and then washed three times with 1 mM EDTA to remove nonspecifically bound copper. The pellet was digested with 50 μL of 70 % *w*/*v* nitric acid at 65 °C for 12 h. The samples were then run at 20-fold dilution for ICP-MS analysis.

### 2.3. Transmission Electron microscopy (TEM) sample preparation

The specimens were transferred onto specimen carriers and high pressure frozen in the Leica EM Ice (Leica Microsystems, Buffalo Grove, IL). For freeze substitution (FS) specimens were transferred into pre-cooled cryogenic vials (Corning Incorporated, Corning, NY 14831, USA) filled with 2.5 % glutaraldehyde in anhydrous acetone. FS was carried out at −90 °C (48 h), −70 °C (24 h), −50 °C (12*h*), −20 °C (12 h), 0 °C (6 h) and RT (1 h). Media was exchanged at −20 °C with 2 % osmium tetroxide in anhydrous acetone. After the samples were rinsed in anhydrous acetone (2 times 15 min), they were infiltrated by mixtures of acetone and EMBed812 epoxy resin (2:1, 1:1, 1:2) and finally pure resin for at least 3 h, each step. The embedded samples were then polymerized in EMBed812 resin for 48 h at 60 °C. Ultrathin sections were sectioned with a ultramicrotome (Leica EM UC7) and post-stained with 2 % lead citrate and 1 % uranylic acetate for 5 and 15 min, respectively. Sections were imaged at 60 kV in STEM mode with a transmission electron microscope (Spectra 300, FEI Company, Hillsboro, OR).

### 2.4. Total RNA library preparation

The RNA library was prepared by Macrogen as described previously [35]. Whole-cell RNA was treated with DNase to eliminate DNA contamination. mRNA purification kit was used to extract mRNA with poly-A tail. Ribo-zero RNA removal kit was used to purify RNA of interest for non-coding RNAs. Fragmented RNA was reverse transcribed into cDNA. Both ends of the cDNA fragments were ligated with adapters. After amplifying fragments by PCR, fragments with insert sizes between 200 and 400 bp were selected. For pair-end sequencing, both ends of the cDNA were sequenced by the read length.

### 2.5. RNA sequencing

cDNA libraries were sequenced by Macrogen with Illumina HISeq platform. To map cDNA fragments, NCBI R64 was used as a reference genome. To reduce biases in analysis, artifacts such as low-quality reads, adapter sequence, DNA contaminant, or PCR duplicates were removed. Trimmed reads were mapped to reference genome with HISAT2. After the read mapping, Stringtie was used for transcript assembly. Expression profiles are represented as read count and normalization value which is based on transcript length and depth of coverage. The FPKM (Fragment per Kilobase of transcript per Million mapped reads) is used as a normalization value.

### 2.6. Generating clustered heatmap of samples

The pairwise correlation between RNA-seq samples across replicates and conditions were calculated using a Pearson correlation. The values in the cells represent the correlation between the sample on the left axis and the bottom axis. The color is scaled by the magnitude of the correlation. Rows and columns were clustered using the average linkage approach and Euclidean distance. Plots were generated using the Seaborn library in Python [36].

### 2.7. Gene set enrichment analysis (GSEA) for functional interpretation of gene expression changes

Gene Set Enrichment Analysis (GSEA) was performed to gain insights into the functional implications of gene expression changes. The analysis was carried out using R (4.3.1) programming language and the ‘clusterProfiler’ package (version 4.8.2) [37]. Initially, the gene expression dataset was preprocessed, and genes were ranked based on differential expression between experimental conditions. Subsequently, predefined gene sets, including Kyoto Encyclopedia of Genes and Genomes (KEGG) pathways and custom gene sets representing specific biological processes, were used for enrichment analysis. The ‘gseKEGG’ function was employed to assess the enrichment of KEGG pathways. Enrichment results were considered statistically significant at a false discovery rate (FDR) threshold of 0.05 after applying the Benjamini-Hochberg (BH) correction for multiple testing. The results of enriched KEGG pathways in different conditions were visualized using ‘dotplot’ in R.

### 2.8. Calculating gene ontology enrichment for differentially expressed genes

To understand the biological processes influenced by condition-specific NMD regulation we quantified the Gene Ontology (GO) enrichments genes upregulated in NMD-deficient mutants under three environmental conditions. Genes were considered to be upregulated if the comparison between the NMD mutant strain and the wildtype had a fold change greater than 1.5 and a *p*-value less than 0.05.

Three single comparison sets were generated by identifying genes upregulated in only one condition: CM (normal growth), LC (low copper), or 100uM Cu (high copper). Four shared comparison sets were also generated, considering genes that were upregulated in the following combinations of conditions: LC/CM, LC/100uM, CM/100uM, and all conditions. For each comparison set, an overrepresentation analysis was run using WebGestalt with two ontologies (GO Biological Process, GO Molecular Function) and the KEGG pathway database [38–41]. For each GO term in the ontology, we compute enrichment of genes annotated with that term relative to a set of reference genes. We used the set of protein-coding genes in the *S. cerevisiae* genome with RNA-sequencing as our reference set and determined significance with a FDR threshold (q value) of 0.05. We corrected for multiple comparisons using the Benjamini-Hochberg procedure. When many similar terms were present, we applied affinity propagation to cluster the gene sets using the APCluster package in R [42]. We used the clustered terms to label the visualizations.

### 2.9. RNA methods

Total *S. cerevisiae* RNA was used for all mRNA steady-state analysis. Northern blots were conducted, probed and phosphorimaged using a Typhoon Phosphorimager (Amersham Pharmacia Biotech, Inc.). Northern blots were probed with *CYH2* as an NMD control [33]. *CTR1* mRNA served as the low copper control due to its role as a high-affinity copper transporter of the plasma membrane, which is upregulated in response to copper deficiency. *CUP1* mRNA, encoding a metallothionein that binds copper, was utilized as the high copper control. *CUP1* mRNA is induced by increased copper levels. *SCR1* RNA was used as the loading control for all northern blots to ensure equal RNA loading across samples. Quantification of northern blots were performed using Image-Quant^™^TL 10.1(Cytiva) software.

## 3. Results

### 3.1. NMD *(upf1Δ)* mutants accumulate higher amounts of cellular copper when yeast cells are grown under normal copper levels

Prior to growing wild type and NMD mutants (*upf1*Δ) yeast cells in different growth conditions, we measured the concentration of copper in complete minimal (CM) media, low copper (CM containing 100 μM of the extracellular copper chelator BCS) and CM containing 100 μM copper. We found that CM contains 0.06 (± 0.02) μM copper, while there was no detectable copper in the low copper media (0.00 (± 0.03) μM) (Table 1). The highest concentration of copper (75 (± 0.42) μM) was found in the CM containing 100 μM copper (Table 1). Comparing the growth of wild type and NMD mutant strains, we found no difference in growth between the two strains when the cells were grown in CM or CM containing 100 μM copper (Supplementary Fig. 1A). Both wild type and NMD mutants (*upf1*Δ) grew slower in low copper relative to CM due to deficiency of copper (Supplementary Fig. 1B). Slow growth of yeast cells grown in 5 μM BCS has been previously reported in other studies [43].

**Fig. 1.**
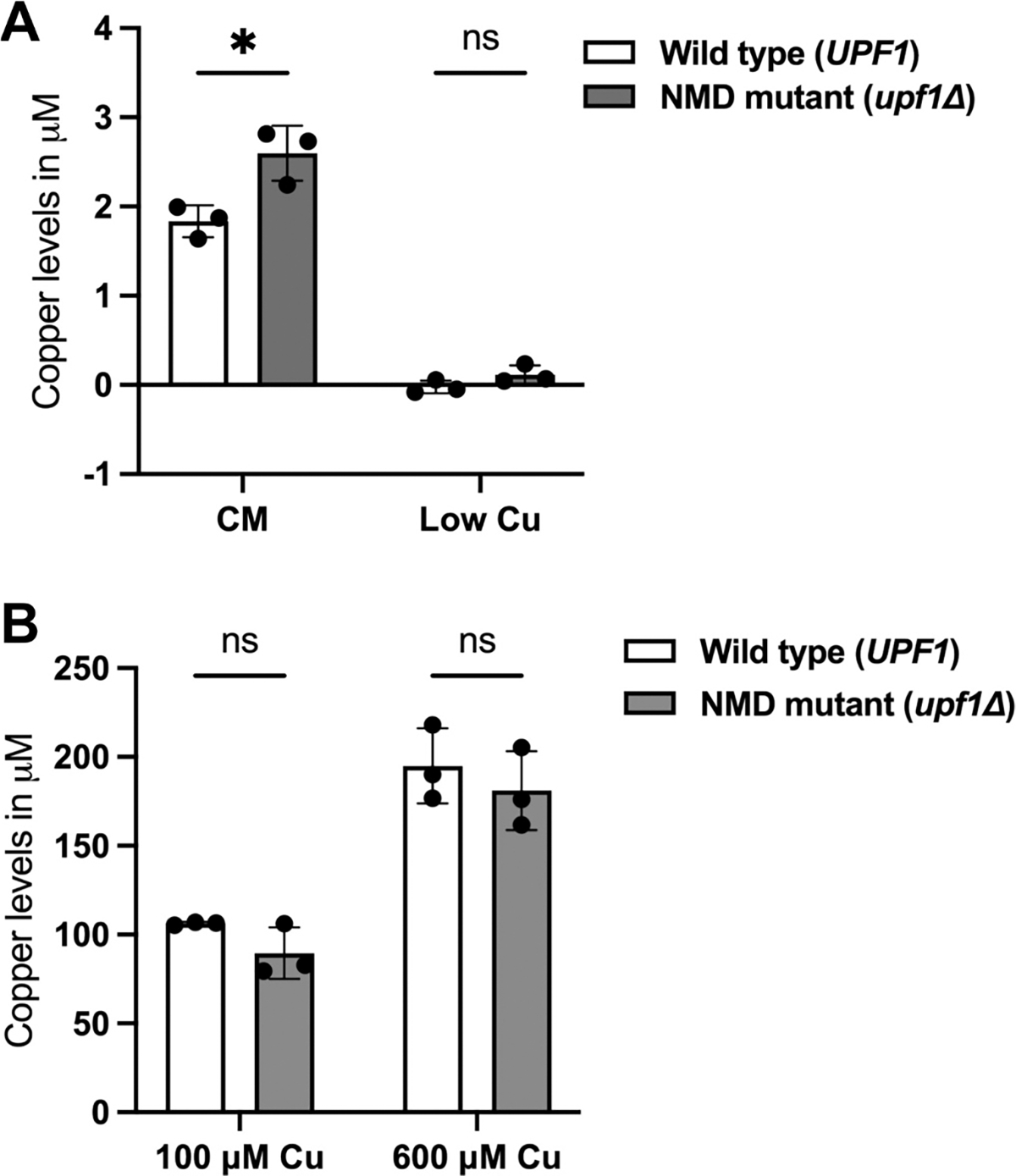
Total amounts of copper levels in wild type and NMD mutants grown in CM and low copper (A), 100 μM copper and 600 μM copper (B). * *p* < 0.05, ns, not significant.

Subsequently, we quantified the total cellular copper levels in wild type and NMD *(upf1Δ)* mutants. Cells were grown in the three conditions above and 600 μM copper for high copper conditions. We included 600 μM copper because we previously found that NMD mutants tolerate high levels of copper above 600 μM compared to wild type strains [34]. NMD mutants accumulated significantly higher levels of copper relative to the wild type strain grown in CM (Fig. 1A). This is likely due to NMD-mediated regulation of low affinity copper transporters *FET4* and *CTR2* [8,26,44]. Copper levels in low copper conditions were undetectable for both yeast strains (Fig. 1A and Table 1). The total cellular copper levels increased as the extracellular copper levels in the growth medium increased from CM to 100 μM and 600 μM copper (Fig. 1A and B).

In our previous study total cellular copper levels were measured along with cytoplasmic copper levels using a β-galactosidase reporter assay [34]. Additionally, vacuolar-associated copper levels were measured [34]. We found that copper levels of 600 μM elicited a physiological response between the wild type and NMD mutant strains [34]. NMD mutants had enhanced growth relative to the wild type strain at 600 μM copper [30,34,44]. Although previous physiological studies demonstrated that NMD mutants were more tolerant of higher levels of copper, there was no significant difference in total cellular copper levels between wild type and NMD mutants grown in 100 and 600 μM copper (Fig. 1B) [34]. However, in our earlier study we found that cytoplasmic copper levels are significantly lower in NMD mutants relative to wild type strains grown in CM containing 600 μM copper [34]. These observations may be attributed to NMD-mediated regulation of mRNAs encoding proteins involved in high copper tolerance, which function in sequestering copper into the vacuole.

Iron, zinc and copper transport are linked. Fet4p is a multicopper oxidase that transports iron, zinc and copper into yeast cells [45]. Thus, we quantified the total cellular levels of iron and zinc in wild type and NMD *(upf1Δ)* mutants grown in CM, low copper, CM containing 100 μM copper, and 600 μM copper. We found that under low copper conditions zinc levels were upregulated in both wild type and NMD mutants compared to CM, low copper and high copper (Supplementary Fig. 2A). The upregulation of zinc could be attributed to slightly elevated zinc levels in the low copper media (Supplementary Fig. 2C). However, there was no difference in zinc accumulation levels between the wild type and the NMD mutant strains. Under low copper conditions there was a reduction in the levels of iron in the cells compared to CM, and high copper (Supplementary Fig. 2B).

### 3.2. Dilated endoplasmic reticulum (ER) observed in wild type strains grown in high copper conditions is lost in NMD (*upf1Δ*) mutant strains

**Fig. 2.**
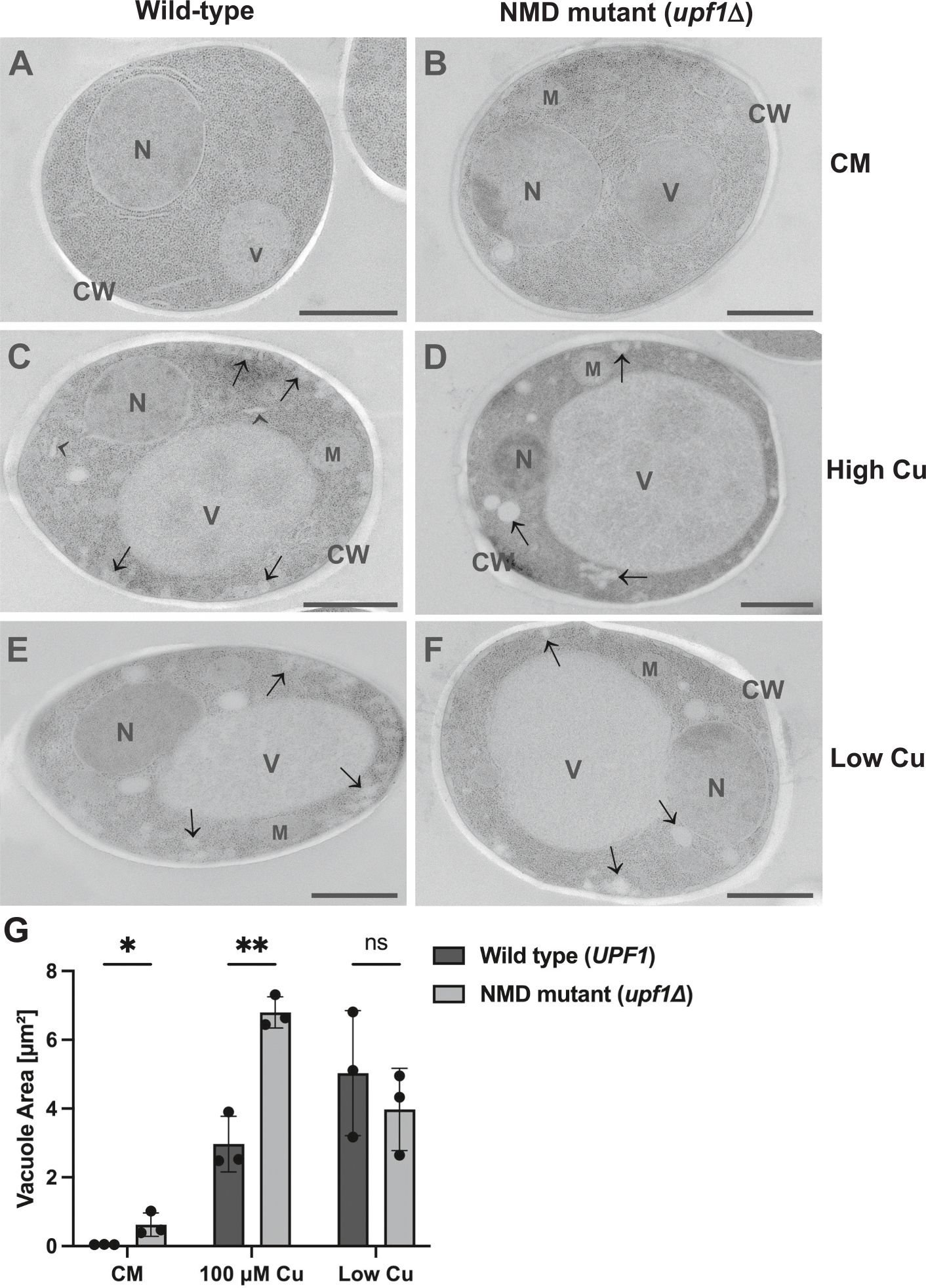
Representative TEM micrographs of high-pressure frozen yeast cells from wild type (A, C, E) and NMD mutant (B, D, F) strains. Images show CM control cells (A, B) with a nucleus (N) containing hetero- and euchromatin, a small vacuole (V), and mitochondria (M). Yeast cells treated with elevated copper concentration of 100 μM (C, D) contained a large central vacuole (V), nuclei (N), and mitochondria (M). These cells also contained a large number of small vacuoles and vesicles which accumulated specifically along the cell membrane and throughout the cytosol of the cells (arrows). Additionally, dilated ER (arrowhead) was commonly found in wild type cells throughout the cytosol. The ultrastructure of yeast cells grown in media with low copper concentrations (E, F) was very similar to cells grown in high copper concentrations. Such cells contained a large central vacuole (V), and a large number of small vacuoles and vesicles along the plasma membrane and inside the cytosol (arrows). The ultrastructure of nuclei (N) and mitochondria (M) in yeast cells grown in media with high (C, D) and low copper concentrations (E, F) did not differ from CM control cells. CW = cell walls. Bars = 1 μm. Representative vacuole area is displayed as bar plot (G). * *p* < 0.05, ** *p* < 0.01, ns, not significant.

**Fig. 3.**
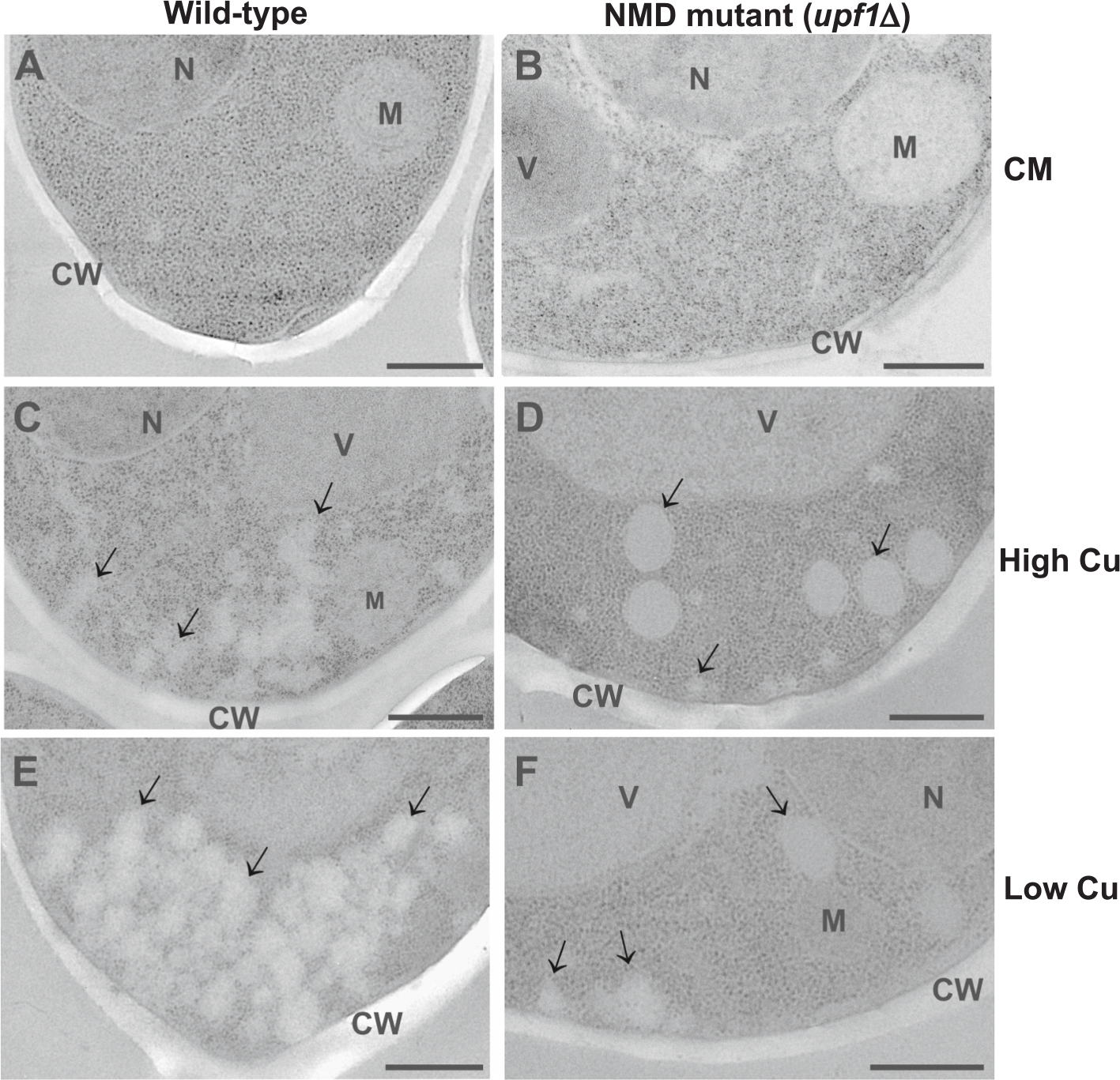
Close ups of TEM micrographs of high-pressure frozen yeast cells from wild type(A, C, E) and NMD mutant (B, D, F) strains. While control cells (A, B) show a dense cytosol without an accumulation of small vesicles/vacuoles inside the cytosol, yeast cells grown in media with high copper (100 μM) and low copper (100 μM BCS) concentrations contained a large number of vesicles and vacuoles along the plasma membrane and inside the cytosol (arrows). CW = cell walls, M = mito-chondria, N = nuclei, V = vacuoles. Bars = 0.5 μm.

To better understand the physiological effects of varying copper levels, wild type and NMD mutant yeast cells grown under CM, 100 μM copper and low copper conditions were observed by transmission electron microscopy (TEM). Observation of wild type and NMD mutant yeast strains grown in CM showed a dense cytosol, with mitochondria, one small vacuole filled with granular material, and well-preserved nuclei with dense hetero- and euchromatin (Fig. 2A, B and 3A, B). When grown under 100 μM copper, both yeast strains contained a dense cytosol with one vacuole filled with granular material which was much larger than the ones in cells grown under normal growth conditions (Fig. 2C, D and 3C, D). The enlarged size of vacuoles may be due to yeast cells importing excess copper into the vacuole to maintain copper homeostasis. Examination of multiple images showed that the enlarged vacuoles in the NMD mutants were larger than those in the wild type cells in CM and 100 μM copper, but not in low copper conditions (Fig. 2G). Other cellular compartments such as nuclei and mitochondria appeared similar when compared to the CM control. Additionally, examination of multiple TEM images showed that the cell wall was slightly thicker in both wild type and NMD mutants grown under low copper conditions but not in CM or 100 μM copper (Supplementary Fig. 3).

Cells from both yeast strains accumulated large amounts of vesicles throughout the cytosol and specifically along the plasma membrane when grown in media with elevated copper levels (Fig. 3C, D). We do not assume that vesicles in the cytosol of cells in Figs. 2 and 3 are dilated ER. Dilated ER (labeled with arrowheads) can only be found in Fig. 2C while vesicles (labeled with arrows) can be found throughout cells displayed in Figs. 2C–F, and Fig. 3C–F. We specifically point out irregularly shaped dilated ER with arrowheads in Fig. 2C in cells with a functional NMD pathway treated with high amounts of copper. None of the other cells show dilated ER. Dilated ER strands (labeled with arrowheads) in Fig. 2C contain ribosomes (dark dots) on the outside of both membranes and their shape differs significantly from the vesicles (labeled with arrows) and can therefore be identified as rough ER. All other vesicles are labeled with arrows and don’t contain ribosomes on the outside of the membrane and are roundish shaped. Dilated ER were not observed in the NMD mutants grown in 100 μM copper or in cells grown in low copper conditions. Interestingly, the ultrastructure of both yeast strains grown in low copper was comparable to cells grown in high copper. These cells contained a large central vacuole filled with granular material in the dense cytosol, mitochondria, a well-preserved nucleus, and many small vacuoles and vesicles along the plasma membrane (Fig. 2E, F and 3E, 2F).

### 3.3. Correlation coefficient analysis between samples shows gene expression difference from low copper conditions

To investigate how copper affects NMD function genome-wide in yeast cells, we performed bulk RNA-seq on steady state levels of mRNA. RNA was extracted from wild type (*UPF1*) and NMD mutant (*upf1Δ*) strains grown in CM, CM media containing 100 μM copper (high copper) and low copper conditions (100 μM BCS).

A total number of 6445 genes were identified from the original raw data. We calculated the pairwise correlation between RNA-seq samples across replicates and conditions using a Pearson’s correlation of counts (Supplementary Fig. 4A). The values in the cells represent the correlation between the sample on the right axis and the bottom axis. The color is scaled by the magnitude of the correlation. Rows and columns were clustered using the average linkage approach and Euclidean distance. We generated plots using the Seaborn library in Python [36]. The wild type samples in low copper condition were most distinct from other samples in different conditions.

To identify NMD regulated transcripts in the different growth conditions, we used 1.5-fold change (FC) as a cutoff for significant change. *P* value was calculated by nbinomWaldTest using DESeq2. Up and down regulated transcripts were required to be statistically significant at *p* value of <0.05 and show a ≥ 1.5-fold average up-regulation or down-regulation in expression in NMD mutants. The similarity between transcripts with ≥1.5-fold change in NMD mutant relative to wild type strain in different environmental conditions were obtained using a Pearson’s correlation of the counts (Supplementary Fig. 4B). Comparing other growth conditions with normal growth condition, this matrix indicated that transcripts with ≥1.5-fold change in the low copper condition were significantly different from CM. Meanwhile, genes with ≥1.5-fold change in NMD mutant relative to wild type under high copper had a medium correlation with CM.

### 3.4. Copper globally influences the regulation of gene expression by the NMD pathway

**Fig. 4.**
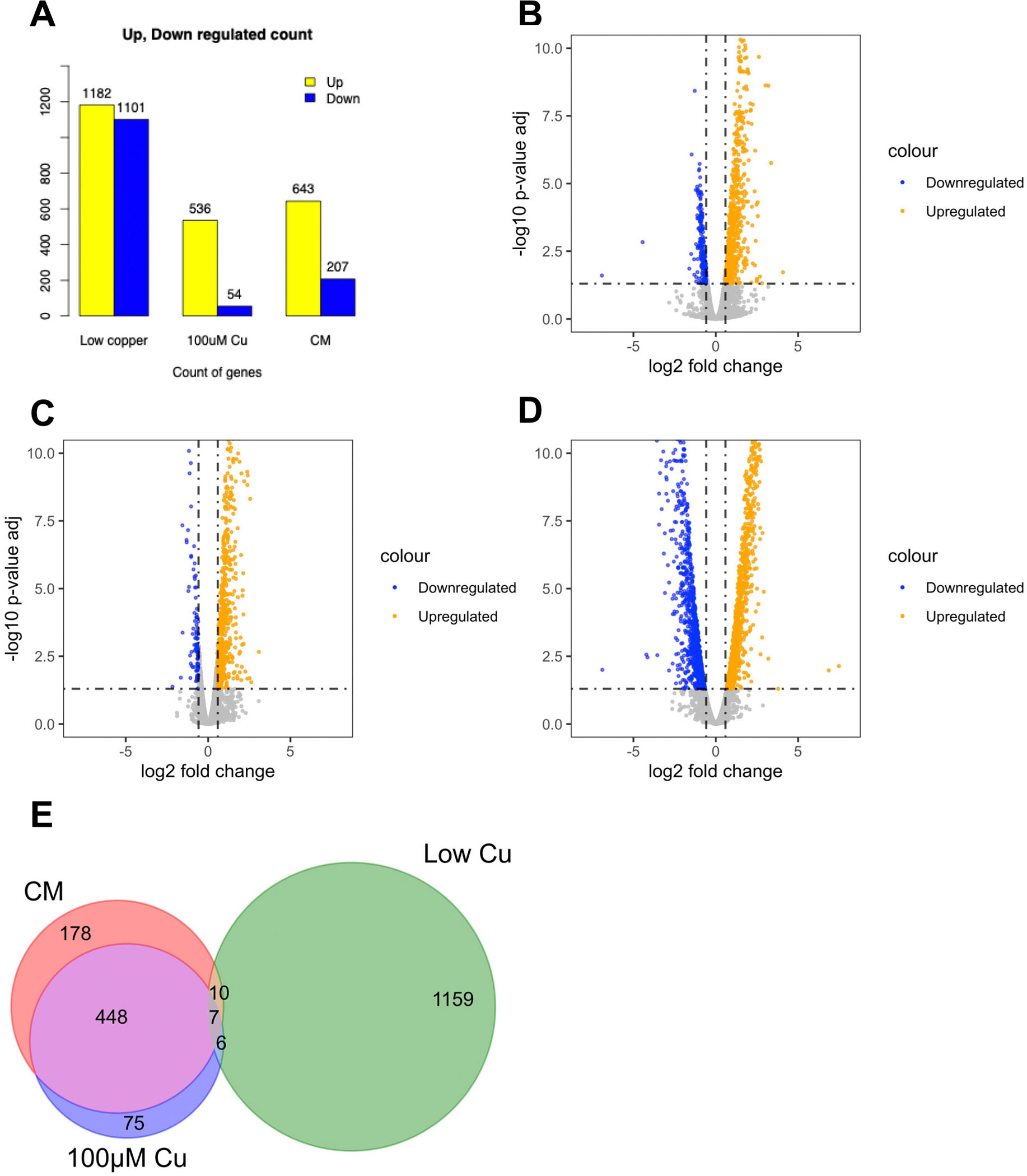
Number of upregulated and downregulated genes in NMD mutant with fold changes of mRNA expression levels more than or equal to 1.5 and p < 0.05 in conditions of CM, low copper and 100 μM copper (A). Fold changes (*upf1Δ /UPF1*) of mRNA expression levels in CM (B), 100 μM copper (C) and low copper (D) with RNA-seq reveals sensitivity to NMD. NMD-sensitive mRNAs exhibit ≥1.5-fold change in steady-state levels in *upf1Δ* (statistically significant at a false discovery rate < 0.05). Venn diagram of number of genes upregulated in NMD mutants in different environmental conditions (E).

Up and down regulated counts of genes in CM, high copper (100 μM copper) and low copper conditions (100 μM BCS) are depicted in Fig. 4A. Comparing wild type and NMD mutant (*upf1Δ*) strains in CM, 643 genes out of 6445 genes were identified whose mRNA expression increased ≥1.5-fold in the NMD mutant (*upf1Δ*) relative to wild type, while 207 transcripts were down-regulated ≥1.5-fold in the NMD mutant (*upf1Δ*) relative to wild type cells (Fig. 4A and B).

The number of transcripts identified under normal growth conditions is consistent with previous studies that identified 5–10 % of the *S. cerevisiae* transcriptome as NMD substrates. We compared our results with two previous genome-wide studies of NMD-regulated transcripts [46,47]. These two studies performed RNA sequencing on the yeast transcriptome comparing wild type strains and NMD mutants, though they grew the cells in rich media (YEPD) instead of CM as normal growth conditions. In this study CM was used as normal growth condition to compare with the low copper and high copper conditions. Transcripts upregulated in the NMD mutant (*upf1Δ*) strains at raw *p* value of <0.05 and ≥ 1.5-fold change were sorted as NMD substrates from Celik et al. 2017 and Malabat et al. 2015. Celik et al. 2017 identified 1304 transcripts that were NMD substrates. 38 % of transcripts identified from Celik et al. 2017 overlapped with transcripts identified in this study. Malabat et al. 2015 identified 1271 as NMD targets, and 36 % of them were identified in this study (Table 2).

Our analysis also identified 536 genes encoding transcripts whose expression increased ≥1.5-fold, and 54 transcripts of which expression decreased ≥1.5-fold in NMD mutant versus wild type strains in high copper conditions (100 μM copper) (Fig. 4A and C). Notably, in low copper conditions (100 μM BCS), 1182 transcripts were up-regulated ≥1.5-fold and 1101 transcripts were down-regulated ≥1.5-fold in NMD mutant versus wild type strains (Fig. 4D). The changes in the number of genes encoding transcripts that were upregulated in the NMD mutant relative to wild type strain indicate that copper affects NMD regulated mRNAs globally. Interestingly, genes downregulated in NMD mutants relative to wild type strains indicate that these genes encoded transcripts that accumulated to higher levels when NMD is functional. This is likely due to transcriptional repressors being regulated by NMD. In NMD mutant cells, these transcriptional repressors are elevated and subsequently repress gene expression of their target genes. Thus, gene expression may be modified by NMD depending on environmental conditions. In all three conditions tested, there were more genes upregulated than downregulated in NMD mutants relative to the wild type strains (Fig. 4A).

To further characterize the transcripts that vary in NMD regulation when environmental conditions change, we compared transcripts upregulated in NMD mutants in low copper and high copper conditions with those upregulated in CM. Seven transcripts accumulated ≥1.5-fold higher in NMD mutants relative to wild type strains in all environmental conditions tested. In addition, 178 genes encoded transcripts that accumulated ≥1.5-fold in NMD mutants relative to wild type cells only in CM. 75 encoded transcripts that were up-regulated ≥1.5-fold in NMD mutants versus wild type strains only in copper replete conditions (100 μM copper). The expression of 1159 transcripts increased ≥1.5-fold only in copper deplete conditions (100 μM BCS) (Fig. 4E). An overlap of 448 genes was observed between the transcripts that are upregulated in both CM and high copper, while the transcripts that are upregulated in low copper are significantly distinct from the other two conditions.

### 3.5. Gene set enrichment analysis reveals differentially expressed transcripts and pathway enrichment variability in different copper levels

**Fig. 5.**
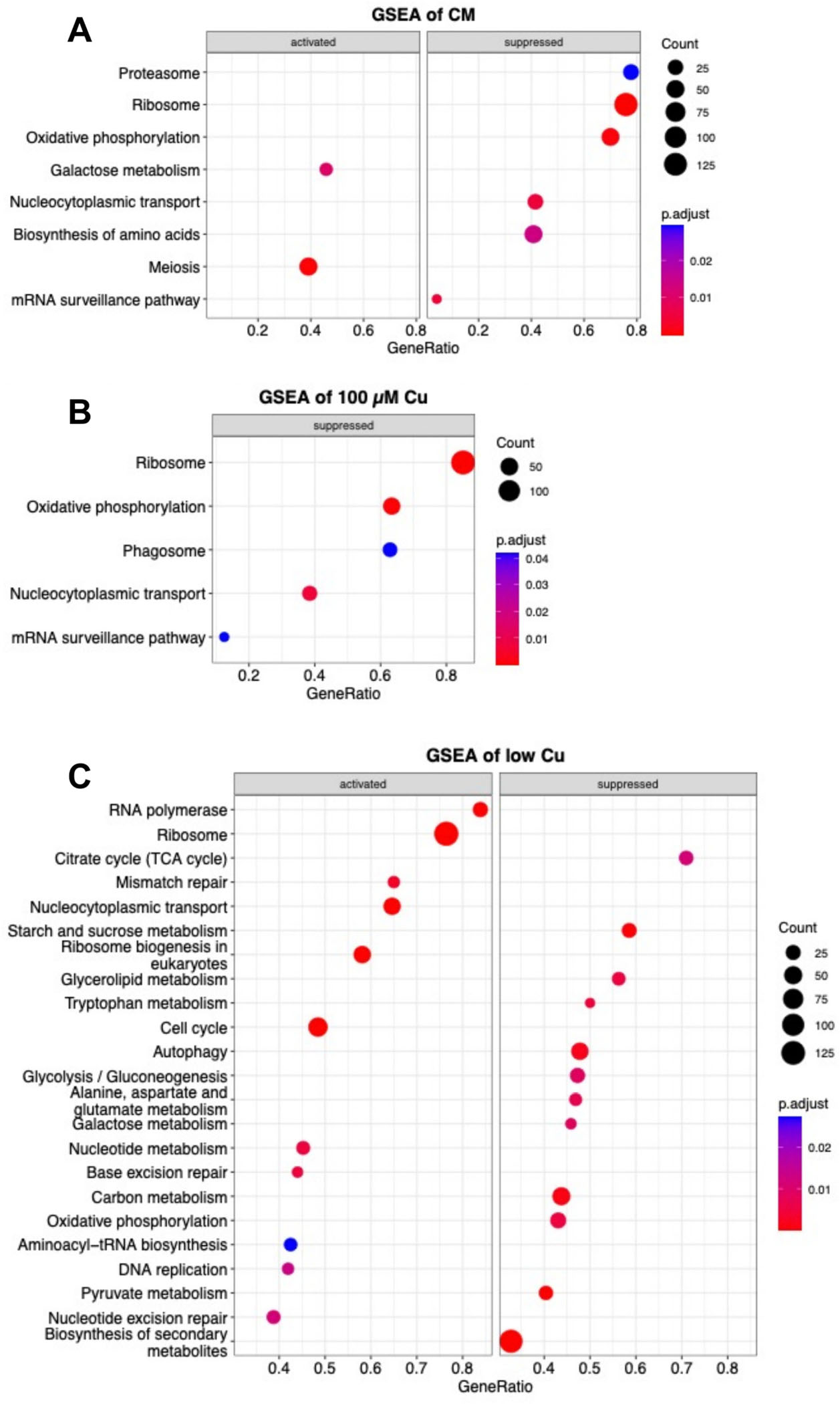
Gene Set Enrichment Analysis (GSEA) for transcripts ranked by differential expression levels in CM (A), 100 μM copper (B) and low copper (C).

To identify the biological pathways and processes associated with the observed gene expression changes in different copper levels, the gene list was ranked based on the differential expression between wild type and NMD mutant strains. After ranking, the biological pathways enrichment was determined within the ranked list of genes. Suppression of oxidative phosphorylation was observed in all three growth conditions tested (Fig. 5). In normal growth conditions, meiosis and galactose metabolism were activated (Fig. 5A). The activation of meiosis in NMD mutants is consistent with previous identification of genes involved in meiosis as NMD substrates [8]. The mRNA surveillance pathway is suppressed in both CM and 100 μM copper. This could be because of *UPF1* being knocked out in the NMD mutant strain. In high copper conditions, only suppressed pathways were observed (Fig. 5B).

Under low copper conditions, the activated and suppressed biological pathways were more altered compared to CM and 100 μM copper. The activation of RNA polymerase and ribosomes in NMD mutant cells demonstrated that transcripts involved in transcription and translation were regulated by NMD in low copper conditions (Fig. 5C). Further, activation of three DNA repair pathways, including mismatch repair, base excision repair and nucleotide excision repair was observed under low copper.

### 3.6. Functional categories of condition-specific upregulated genes that are significantly enriched

To understand the biological processes influenced by condition-specific NMD regulation we quantified the Gene Ontology (GO) enrichments genes upregulated in NMD-deficient mutants under the three environmental conditions. We considered transcripts to be upregulated if the comparison between the NMD mutant strain and the wild type had a fold-change greater than 1.5 and a false discovery rate less than 0.05.

**Fig. 6.**
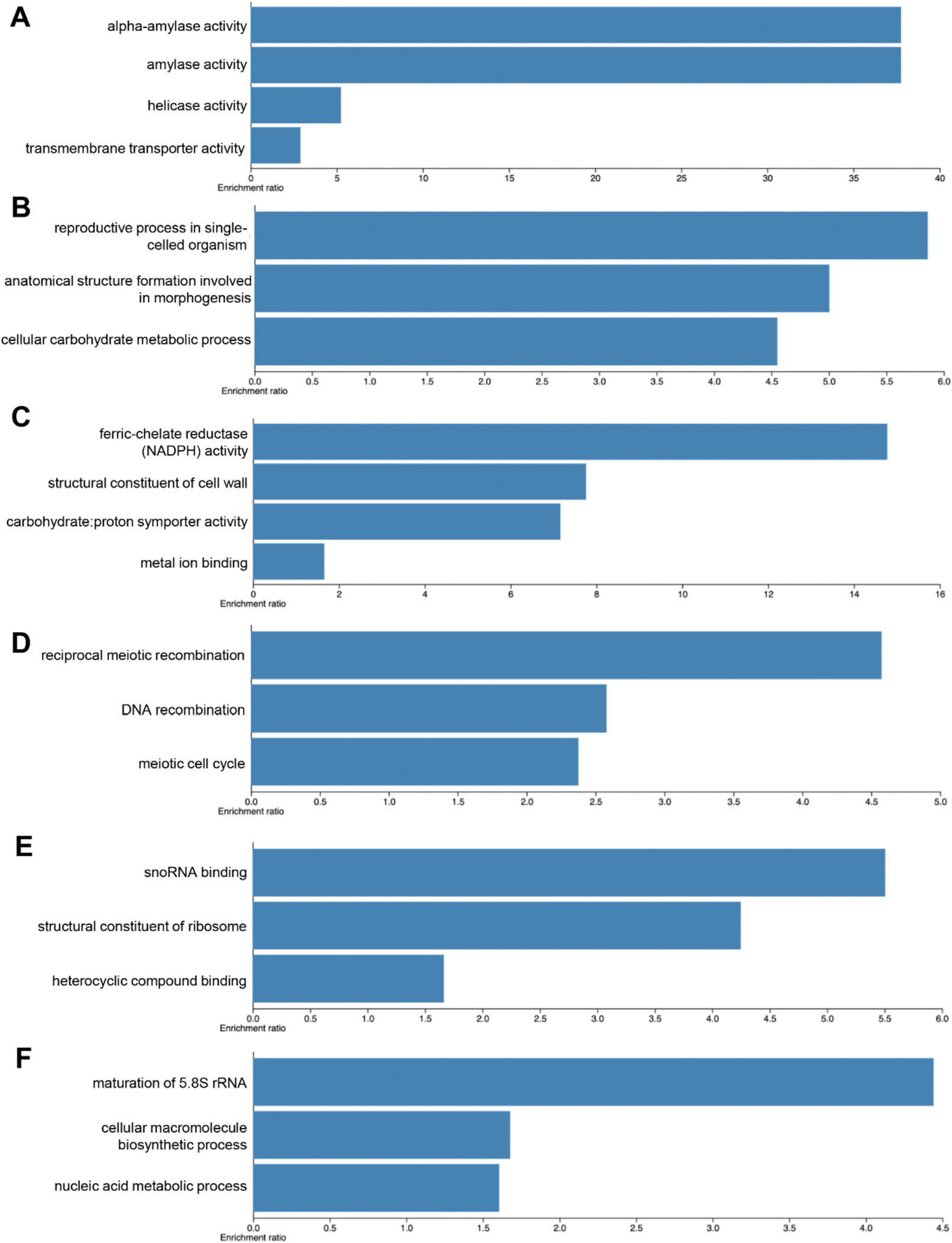
Gene Ontology (GO) enrichments for Molecular Function terms (A) and Biological Process terms (B) for genes upregulated in NMD mutant (*upf1*Δ) under CM only. GO enrichments for Molecular Function terms (C) and Biological Process terms (D) for genes upregulated in NMD mutant (*upf1Δ)* under both CM and 100 μM copper conditions. GO enrichments for Molecular Function terms (E) and Biological Process terms (F) for genes upregulated in NMD mutant (*upf1Δ)* in low copper only. Gene sets were clustered for visualization using affinity propagation.

Under CM only, genes upregulated in NMD mutants were enriched for Molecular Function (MF) terms related to transporter and catalytic activity including helicase activity (q = 0.002) and transmembrane transporter activity (q = 0.002) (Fig. 6A; Supplementary Table 1). These genes were enriched for additional Biological Process terms, including those related to reproductive processes (q = 8.3E-6), morphogenesis (q = 2.1E-5), maltose metabolic process (q = 2.4E-5), and meiotic cell cycle (q = 2.41E-5) (Fig. 6B; Supplementary Table 2). These results were consistent with KEGG pathway enrichments for galactose metabolism (q = 2.8E-4) and starch and sucrose metabolism (q = 0.004).

For genes upregulated in both the normal growth and high copper conditions, we found molecular function enrichments specifically for DNA binding (q = 0.001), metal ion (q = 0.001) and cation binding (q = 0.001), and we also observed significant enrichment for ferric-chelate reductase activity (q = 7.9E-4) and structural constituent of the cell wall (q = 1.44E-7) (Molecular Function, Fig. 6C; Supplementary Table 3). Like the normal growth condition alone, we found enrichment for Biological Process terms related to cell cycle and meiosis with top enrichments for the genes shared by the normal and high copper conditions including DNA recombination (q = 4.27E-5) and meiotic cell cycle (q = 4.27E-5) (Fig. 6D; Supplementary Table 4).

Genes upregulated in the NMD mutant *(upf1Δ),* only under low copper conditions were enriched for molecular functions related to organic cyclic compound binding, heterocyclic compound binding, nucleic acid binding, RNA binding and ribosome structure including translation factor activity, RNA binding (q = 6.02E-12) and structural constituent of ribosome (q < 2.2E-16) (Molecular Function, Fig. 6E and Supplementary Table 5). These genes were also enriched for Biological Process terms related to nucleic acid metabolic process and macromolecule synthesis including RNA metabolic process (q < 2.2E-16), cellular nitrogen compound biosynthesis process (q < 2.2E-16) (Fig. 6F and Supplementary Table 6). We found no significantly enriched terms for genes upregulated only under high copper conditions, for the genes shared between the low copper condition and normal growth conditions, or for genes upregulated in NMD mutants in all three conditions. However, for the subset of genes upregulated in both high and low copper conditions, we find significant enrichment for terms related to ferric-chelate reductase activity (q = 0.01) and oxidoreductase activity (q = 0.01) (Supplementary Fig. 5).

Additional gene expression analyses were done on the wild type strain in normal growth versus low copper conditions, and wild type grown in normal growth versus high copper. In normal growth versus low copper conditions, we found 423 differentially expressed genes. These genes were enriched for terms related to transporter activity and ion binding, in addition to various metabolic and biosynthetic processes in the Biological Process and Molecular Function ontologies (Supplementary Table 7). These differentially expressed genes were also enriched in storage (*p* = 6.88 × 10–8) and lytic vacuoles (p = 6.88 × 10–8) and the vacuolar membrane (*p* = 8.24 × 10–4) from the Cellular Component ontology.

In normal growth versus high copper conditions, we identified one significantly enriched term from the Biological Process ontology (vitamin metabolic process, *p* = 1.83 × 10–4) and none from the Molecular Function or Cellular Component ontologies after multiple testing correction (Supplementary Table 8). However, we did not observe many genes that were differentially expressed in these conditions (*n* = 13). Thus, there is limited statistical power and differences are based on expression of a very small number of genes (~1–2 genes.

### 3.7. Validation of transcripts differentially regulated by NMD in specific conditions

**Fig. 7.**
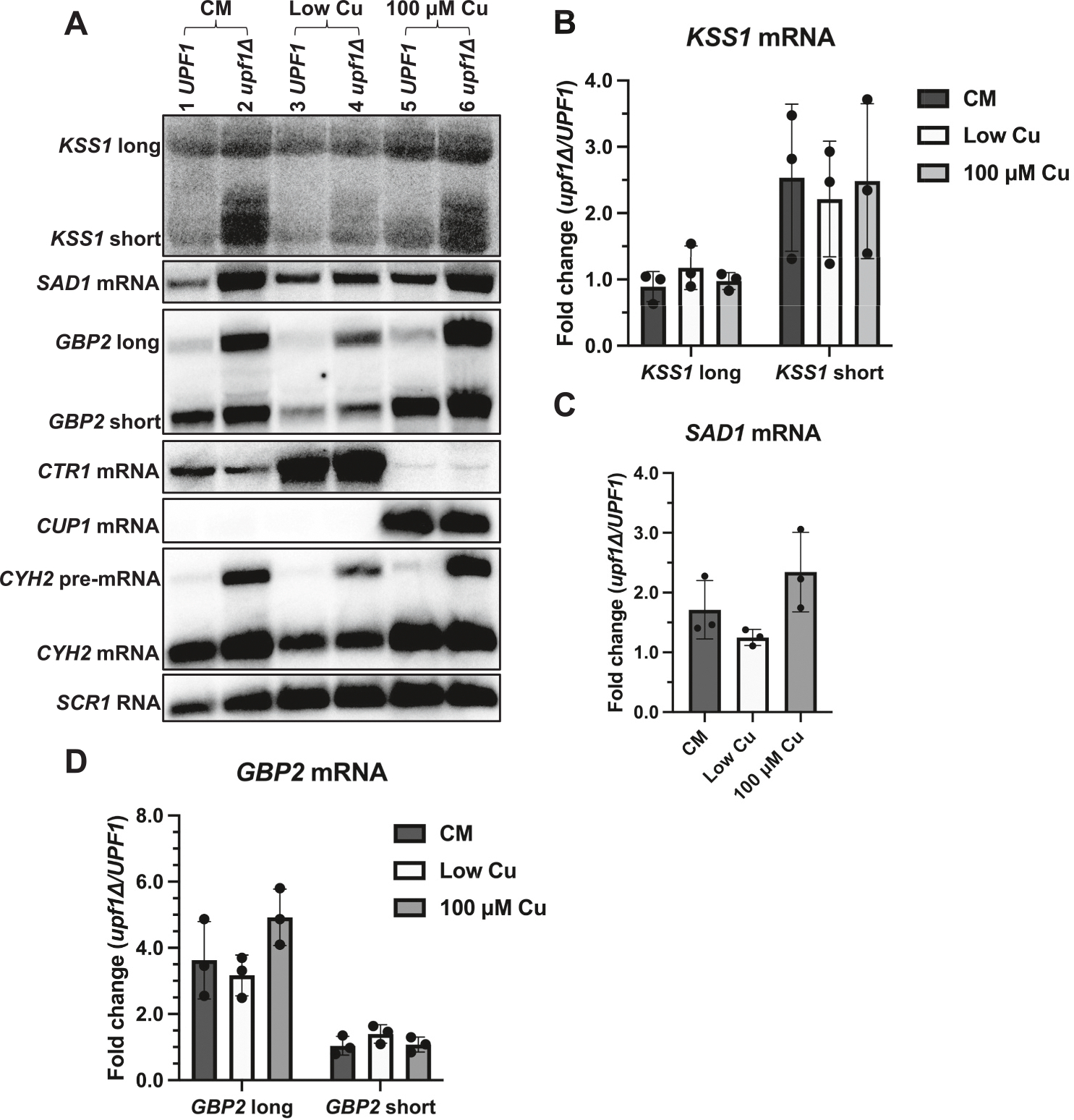
Representative mRNA steady-state accumulation levels of *KSS1*, *SAD1*, and *GBP2* mRNAs (A). The northern blots were probed with DNA specific to *KSS1*, *SAD1*, and *GBP2* open reading frames (ORFs). Fold changes (*upf1Δ/UPF1*) of *KSS1* mRNA (B), *SAD1* mRNA (C), and *GBP2* mRNA(D). *CTR1*, *CUP1*, *CYH2* and *SCR1* were used as controls. The steady-state measurements are an average of three independent experiments represented by spots on the bar graphs.

To validate the conditionally regulated transcripts, three transcripts were selected based on their expression levels in the three growth conditions and analyzed using northern blots (Table 3). All transcripts were detected by probes specific to their open reading frames (ORFs). The mitogen activating protein kinase (MAPK)-*KSS1* was upregulated ~2-fold in NMD mutants in all three conditions based on RNA-Seq. Northern blot analysis of *KSS1* detected two isoforms in all three conditions. Regulation of the shorter *KSS1* mRNA isoforms was NMD dependent in all the tested conditions (Fig. 7A and B). NMD-mediated regulation of the shorter *KSS1* mRNA isoform relative to the long isoform under the conditions tested here could be due to the additional sequences in the 5′ or 3′-UTR of the *KSS1* transcript, containing a stabilizing element that forms an mRNA stabilizing structure. mRNAs containing long 3′-UTR that fold into stabilizing structures have been reported to escape NMD [48]. *SAD1* were upregulated ~2 fold in NMD mutants in CM and ~ 1.7 fold in 100 μM copper based on RNA-Seq analysis. Northern blot analysis of *SAD1* corroborated the RNA-seq analysis (Fig. 7A and C).

*GBP2* mRNA was upregulated ~2-fold in NMD mutants in low copper based on RNA-Seq. Northern blot analysis of *GBP2* detected two mRNA isoforms that were differentially regulated in CM, low and high copper conditions (Fig. 7A and D). Previous studies on *GBP2* have mainly focused on Gbp2 protein [49,50]. We identified two isoforms of *GBP2* mRNAs for the first time. While the RNA seq analysis only identified *GBP2* mRNA upregulated in low copper, it was likely that the RNA-Seq analysis only detected the short isoform of *GBP2* mRNA.

## 4. Discussion

Gene expression and cellular ultrastructure of wild type and NMD mutants are affected by changes in copper levels. Our previous studies showed that NMD regulating eight mRNAs involved in copper homeostasis [28,29,34,44]. Our current transcriptome wide analysis revealed that changes in copper levels lead to widespread changes in regulation of gene expression by NMD. Most natural NMD targets were of low abundance. Further, when yeast cells were grown in low copper conditions, some NMD targets became less abundant and thus difficult to detect.

Yeast vacuoles play a critical role in copper homeostasis [24,43]. Elevated copper levels caused enlargement of the vacuole and an increase in the amounts of vesicles throughout the cytosol and specifically along the plasma membrane. We posit that a larger number of vesicles increased the surface area to volume ratio, leading to enhanced copper uptake from the cytosol into the vesicles in yeast cells under high copper conditions. Additionally, the increase in vacuolar size was more pronounced in NMD mutants relative to the wild type strains in CM and high copper (Fig. 2G). This is likely due to elevated levels of mRNAs encoding proteins involved in copper import into the vacuole [8]. Furthermore, differential regulation of mRNAs encoding ferric chelate reductase by NMD supports accumulation of copper in the vacuole. *FRE2*–*7* mRNAs were regulated by NMD in CM. In high copper conditions *FRE2*–5 and *FRE7* mRNAs were regulated by NMD, *FRE6* which encodes a reductase targeted to the vacuole escapes NMD mediated degradation in these conditions. These suggests that a functional fre6 protein in essential in high copper. Alternatively, the large number of vesicles may also be a result of vacuolar fragmentation and inhibition of vacuole fusion by copper [51]. Enlargement of the vacuole under low copper conditions in both wild-type and NMD mutants was unexpected and could be due to disruption of metal ion homeostatic mechanisms because they are interconnected (Figs. 2E–F). Metal ions such as zinc could be accumulating in the vacuole. Notably, we found that cellular zinc levels were elevated under low copper conditions (Supplementary Fig. 2 A).

Dilated ER observed in wild type yeast cells exposed to elevated copper levels could be caused by accumulation of misfolded protein aggregates [52]. An abnormal ultrastructure of the ER of hepatocytes was previously observed in patients with Wilson’s disease [53]. Wilson’s disease is an autosomal recessive genetic disorder characterized by the accumulation of copper in various organs. Dysfunction of *ATP7B*, a gene encoding a P-type ATPase copper transporter found in the trans-Golgi network, results in Wilson’s disease, which is characterized by excessive copper accumulation in body. The observation of yeast cells with dilated ER in elevated copper levels demonstrated that yeast phenotype was consistent with human cells (Fig. 2C) [53]. We did not observe dilated ER in NMD mutant cells grown in elevated copper conditions, although the total cellular copper levels did not significantly differ between wild type and NMD mutant yeast cells (Fig. 1B). The amount of copper transporters on organelles might be elevated in NMD mutant cells, thus transporting excessive copper into organelles to alleviate ER stress [8,44,54]. Additionally, the gene set enrichment analysis for transcripts in high copper demonstrated suppressed ribosome metabolic pathways, indicating decreased protein translation (Fig. 5B).

Research has investigated the intricate regulation of transcripts by NMD, with a particular emphasis on elucidating the functions and categorization of genes targeted by NMD. Specifically, a previous study examining the impact of NMD on gene expression in yeast organized the functional relationships of NMD targets into two categories. One category was genes that were affecting chromosome structure and behavior, and the second category was genes that affect cell surface and environmental interactions [8]. In our study, the functional categories of genes upregulated in NMD mutants in CM corroborate these functional categories. Functional categories of genes in CM fall in transmembrane transporter activity and the activation of meiosis regulating chromosome structure and behavior (Fig. 5A).

Additional categories were upregulated in NMD mutants in our study in both CM and high copper conditions. These categories included structural constituents of the cell wall, metal ion binding and DNA binding and recombination. Copper affects DNA damage [55]. Additionally, NMD regulates mRNAs involved in DNA homologous recombination. These mRNAs include *RAD51*, *RAD54*, *RAD55* and *RAD57* [56]. In our study, we found differential NMD regulation of *RAD51* and *RAD57*. *RAD57* is an NMD target in CM and 100 μM copper, but not in low copper. *RAD51* was downregulated in 100 μM copper in NMD mutants but not in CM, or low copper. Enrichment of structural constituents of the cell wall and metal ion binding functional categories highlight the fact that NMD plays a significant role in how cells respond to changes in environmental conditions.

The distinct categories enriched specifically in low copper, resulting in slow growth may be attributed to copper deficiency indirectly affecting ribosome recycling. The essential iron-sulfur (Fe–S) cluster containing RNase L inhibitor (Rli1) stimulates translation termination and ribosome recycling [57–59]. Impairment of the human homolog of Rli1, ABCE1, resulted in movement of ribosomes into mRNA 3′ UTRs, where they displace exon junction complexes (EJCs), inhibiting NMD [60]. The activity of Rli1/ABCE1 requires iron-sulfur (Fe–S) cluster biogenesis. Notably, iron is limited in copper limited conditions because the multicopper oxidase FET3 which is involved in iron uptake requires copper to function [61]. We found reduced iron levels in wild type and NMD mutants grown in low copper (Supplementary Fig. 2B). Therefore, copper deficiency could indirectly impact the NMD pathway by the activity of Rli1/ABCE1.

Genes upregulated in both normal growth and high copper conditions illustrated that NMD plays a regulatory role in metal ion binding when copper was sufficient. For instance, previous literature demonstrated that ferric chelate reductase was required for iron uptake in *S. cerevisiae* [62]. The enrichment of ferric chelate reductase activity was observed in normal and high copper conditions, indicating copper was essential for iron uptake. These findings underscored the interconnectedness of metal ion homeostasis and suggested that NMD could be crucial in maintaining this balance under varying copper levels. Ultimately, this study highlights the importance of environmental conditions, like metal ions having a widespread effect on regulation of gene expression by NMD.

## 5. Conclusions

Environmental stresses/conditions influence the sensitivity of transcripts to NMD, causing adaptation to the environmental stresses and resulting in physiological consequences to cells.

## Supplementary Material

1

2

3

4

5

6

7

8

9

## Figures and Tables

**Table 1:** Copper content in growth media and in wild type and NMD mutants. Copper levels are reported in μM ± standard deviation.

Growth media (copper levels)	Wild type (*UPF1*)	NMD mutant (*upf1Δ*)

Complete minimal (0.06 (± 0.02) μM)	1.8 (± 0.2) μM	2.6 (± 0.3) μM
100 μM BCS* (0.00 (± 0.03) μM)	−0.02 (± 0.07) μM	0.1 (± 0.1) μM
100 μM Cu (75 (± 0.42) μM)	106 (± 0.8) μM	89 (± 14) μM
600 μM Cu	195 (± 21) μM	181 (± 22) μM

* BCS- Bathocuproinedisulfonic acid.

**Table 2:** Overlap of NMD-regulated transcripts identified in this study and in previous studies.

	Celik et al. (2017)	Malabat et al. (2015)

Identified as NMD substrates	1304	1271
Also identified in this study	496	463
% Overlap	38 %	36 %

**Table 3:** Transcripts selected for northern blot validation.

Transcript	Condition identified as NMD target	Gene description

*KSS1*	All conditions	Mitogen-activated protein kinase (MAPK)
*SAD1*	CM and 100 μM Cu	Nuclear protein with roles in splicing and spliceosome assembly. Predicted to enable thiol-dependent deubiquitinase and zinc ion binding activity. Involved in splicesomal complex assembly.
*GBP2*	Low copper	Poly (A) RNA-binding protein involved in translation repression

## Data Availability

The NCBI BioProject accession number for the RNA-seq data presented in this paper is PRJNA1059649. https://www.ncbi.nlm.nih.gov/sra/?term=PRJNA1059649.

## Appendix A. Supplementary data

Supplementary data to this article can be found online at https://doi.org/10.1016/j.ygeno.2025.111020.

## Abbreviations:

ER: Endoplasmic Reticulum
UTR: Untranslated Region
ROS: Reactive Oxygen Species
CM: Complete Minimal
GO: Gene Ontology
BP: Biological Processes
MF: Molecular Function
BCS: Bathocuproinedisulfonic acid
TEM: Transmission Electron Microscopy
FC: Fold Change
MAPK: Mitogen Activating Protein Kinase
ICP-MS: Inductively Coupled Plasma Mass Spectrometry
FPKM: Fragment Per Kilobase of transcript per Million mapped reads
GSEA: Gene Set Enrichment Analysis
KEGG: Kyoto Encyclopedia of Genes and Genomes
FDR: False Discovery Rate

## Glossary

NMD: Nonsense-mediated mRNA Decay
PTC: Premature Termination Codon
ORF: Open Reading Frame
uORF: upstream Open Reading Frame
UPF: Up-Frameshift
SOD1: Superoxide Dismutase
YEPD: Yeast Extract Peptone and dextrose

## References

[1] Maquat LE, When cells stop making sense: effects of nonsense codons on RNA metabolism in vertebrate cells, RNA 1 (5) (1995) 453–465. 7489507 PMC1482424

[2] Leeds P, Peltz SW, Jacobson A, Culbertson MR, The product of the yeast *UPF1* gene is required for rapid turnover of mRNAs containing a premature translational termination codon, Genes Dev. 5 (12a) (1991) 2303–2314. 1748286 10.1101/gad.5.12a.2303

[3] Wada M, Lokugamage KG, Nakagawa K, Narayanan K, Makino S, Interplay between coronavirus, a cytoplasmic RNA virus, and nonsense-mediated mRNA decay pathway, Proc. Natl. Acad. Sci. USA 115 (43) (2018) E10157–E10166. 30297408 10.1073/pnas.1811675115 PMC6205489

[4] Leon K, Ott M, An ‘Arms Race’ between the nonsense-mediated mRNA decay pathway and viral infections, Semin. Cell Dev. Biol. 111 (2021) 101–107. 32553580 10.1016/j.semcdb.2020.05.018 PMC7295464

[5] Zhao Y, Ye X, Shehata M, Dunker W, Xie Z, Karijolich J, The RNA quality control pathway nonsense-mediated mRNA decay targets cellular and viral RNAs to restrict KSHV, Nat. Commun. 11 (1) (2020) 3345. 32620802 10.1038/s41467-020-17151-2 PMC7334219

[6] Medghalchi SM, Frischmeyer PA, Mendell JT, Kelly AG, Lawler AM, Dietz HC, Rent1, a trans-effector of nonsense-mediated mRNA decay, is essential for mammalian embryonic viability, Hum. Mol. Genet. 10 (2) (2001) 99–105. 11152657 10.1093/hmg/10.2.99

[7] He F, Li X, Spatrick P, Casillo R, Dong S, Jacobson A, Genome-wide analysis of mRNAs regulated by the nonsense-mediated and 5′ to 3’ mRNA decay pathways in yeast, Mol. Cell 12 (6) (2003) 1439–1452. 14690598 10.1016/s1097-2765(03)00446-5

[8] Guan Q, Zheng W, Tang S, Liu X, Zinkel RA, Tsui KW, Yandell BS, Culbertson MR, Impact of nonsense-mediated mRNA decay on the global expression profile of budding yeast, PLoS Genet. 2 (11) (2006) e203. 17166056 10.1371/journal.pgen.0020203 PMC1657058

[9] He F, Peltz SW, Donahue JL, Rosbash M, Jacobson A, Stabilization and ribosome association of unspliced pre-mRNAs in a yeast *upf1*-mutant, Proc. Natl. Acad. Sci. USA 90 (15) (1993) 7034–7038. 8346213 10.1073/pnas.90.15.7034 PMC47070

[10] Mitrovich QM, Anderson P, Unproductively spliced ribosomal protein mRNAs are natural targets of mRNA surveillance in C. elegans, Genes Dev. 14 (17) (2000) 2173–2184. 10970881 10.1101/gad.819900 PMC316897

[11] Thompson DM, Parker R, Cytoplasmic decay of intergenic transcripts in Saccharomyces cerevisiae, Mol. Cell. Biol. 27 (1) (2007) 92–101. 17074811 10.1128/MCB.01023-06 PMC1800667

[12] Wang J, Vock VM, Li S, Olivas OR, Wilkinson MF, A quality control pathway that down-regulates aberrant T-cell receptor (TCR) transcripts by a mechanism requiring UPF2 and translation, J. Biol. Chem. 277 (21) (2002) 18489–18493. 11889124 10.1074/jbc.M111781200

[13] Welch EM, Jacobson A, An internal open reading frame triggers nonsense-mediated decay of the yeast *SPT10* mRNA, EMBO J. 18 (21) (1999) 6134–6145. 10545123 10.1093/emboj/18.21.6134 PMC1171677

[14] Peccarelli M, Kebaara BW, Regulation of natural mRNAs by the nonsense-mediated mRNA decay pathway, Eukaryot. Cell 13 (9) (2014) 1126–1135. 25038084 10.1128/EC.00090-14 PMC4187617

[15] Cui Y, Hagan KW, Zhang S, Peltz SW, Identification and characterization of genes that are required for the accelerated degradation of mRNAs containing a premature translational termination codon, Genes Dev. 9 (4) (1995) 423–436. 7883167 10.1101/gad.9.4.423

[16] He F, Jacobson A, Identification of a novel component of the nonsense-mediated mRNA decay pathway by use of an interacting protein screen, Genes Dev. 9 (4) (1995) 437–454. 7883168 10.1101/gad.9.4.437

[17] Kervestin S, Jacobson A, NMD: a multifaceted response to premature translational termination, Nat. Rev. Mol. Cell Biol. 13 (11) (2012) 700–712. 23072888 10.1038/nrm3454 PMC3970730

[18] Zhang X, Kebaara BW, Nonsense-mediated mRNA decay and metal ion homeostasis and detoxification in Saccharomyces cerevisiae, Biometals 35 (6) (2022) 1145–1156. 36255607 10.1007/s10534-022-00450-0 PMC9674712

[19] Karlin KD, Metalloenzymes, structural motifs, and inorganic models, Science 261 (5122) (1993) 701–708. 7688141 10.1126/science.7688141

[20] Linder MC, Hazegh-Azam M, Copper biochemistry and molecular biology, Am. J. Clin. Nutr. 63 (5) (1996) 797S–811S. 8615367 10.1093/ajcn/63.5.797

[21] Bird AJ, Metallosensors, the ups and downs of gene regulation, Adv. Microb. Physiol. 53 (2008) 231–267. 17707146 10.1016/S0065-2911(07)53004-3

[22] Nevitt T, Ohrvik H, Thiele DJ, Charting the travels of copper in eukaryotes from yeast to mammals, Biochim. Biophys. Acta 1823 (9) (2012) 1580–1593. 22387373 10.1016/j.bbamcr.2012.02.011 PMC3392525

[23] De Freitas J, Wintz H, Kim JH, Poynton H, Fox T, Vulpe C, Yeast, a model organism for iron and copper metabolism studies, Biometals 16 (1) (2003) 185–197. 12572678 10.1023/a:1020771000746

[24] van Bakel H, Strengman E, Wijmenga C, Holstege FC, Gene expression profiling and phenotype analyses of S. cerevisiae in response to changing copper reveals six genes with new roles in copper and iron metabolism, Physiol. Genomics 22 (3) (2005) 356–367. 15886332 10.1152/physiolgenomics.00055.2005

[25] Gross C, Kelleher M, Iyer VR, Brown PO, Winge DR, Identification of the copper regulon in Saccharomyces cerevisiae by DNA microarrays, J. Biol. Chem. 275 (41) (2000) 32310–32316. 10922376 10.1074/jbc.M005946200

[26] Johansson MJ, He F, Spatrick P, Li C, Jacobson A, Association of yeast Upf1p with direct substrates of the NMD pathway, Proc. Natl. Acad. Sci. USA 104 (52) (2007) 20872–20877. 18087042 10.1073/pnas.0709257105 PMC2409234

[27] Peccarelli M, Scott TD, Kebaara BW, Nonsense-mediated mRNA decay of the ferric and cupric reductase mRNAs FRE1 and FRE2 in Saccharomyces cerevisiae, FEBS Lett. 593 (22) (2019) 3228–3238. 31322728 10.1002/1873-3468.13545 PMC6878129

[28] Peccarelli M, Scott TD, Steele M, Kebaara BW, mRNAs involved in copper homeostasis are regulated by the nonsense-mediated mRNA decay pathway depending on environmental conditions, Fungal Genet. Biol. 86 (2016) 81–90. 26710966 10.1016/j.fgb.2015.12.011

[29] Murtha K, Hwang M, Peccarelli MC, Scott TD, Kebaara BW, The nonsense-mediated mRNA decay (NMD) pathway differentially regulates COX17, COX19 and COX23 mRNAs, Curr. Genet. 65 (2) (2019) 507–521. 30317392 10.1007/s00294-018-0892-y PMC6420912

[30] Wong A, Lam EM, Pai C, Gunderson A, Carter TE, Kebaara BW, Variation of the response to metal ions and nonsense-mediated mRNA decay across different *Saccharomyces cerevisiae* genetic backgrounds, Yeast 38 (9) (2021) 507–520. 33955055 10.1002/yea.3565 PMC8934196

[31] Zhang X, Kebaara BW, Nonsense-mediated mRNA decay of metal-binding activator MAC1 is dependent on copper levels and 3’-UTR length in Saccharomyces cerevisiae, Curr. Genet. 70 (1) (2024) 5. 38709348 10.1007/s00294-024-01291-9

[32] Wente SR, Rout MP, Blobel G, A new family of yeast nuclear pore complex proteins, J. Cell Biol. 119 (4) (1992) 705–723. 1385442 10.1083/jcb.119.4.705 PMC2289698

[33] Kebaara B, Nazarenus T, Taylor R, Atkin AL, Genetic background affects relative nonsense mRNA accumulation in wild typeand upf mutant yeast strains, Curr. Genet. 43 (3) (2003) 171–177. 12695845 10.1007/s00294-003-0386-3

[34] Wang X, Okonkwo O, Kebaara BW, Physiological basis of copper tolerance of *Saccharomyces cerevisiae* nonsense-mediated mRNA decay mutants, Yeast 30 (5) (2013) 179–190. 23450501 10.1002/yea.2950

[35] Martin JA, Wang Z, Next-generation transcriptome assembly, Nat. Rev. Genet. 12 (10) (2011) 671–682. 21897427 10.1038/nrg3068

[36] Waskom M, Seaborn: statistical data visualization, J. Open Source Software 6 (60) (2021).

[37] Yu G, Wang LG, Han Y, He QY, clusterProfiler: an R package for comparing biological themes among gene clusters, Omics 16 (5) (2012) 284–287. 22455463 10.1089/omi.2011.0118 PMC3339379

[38] Ashburner M, Ball CA, Blake JA, Botstein D, Butler H, Cherry JM, Davis AP, Dolinski K, Dwight SS, Eppig JT, , Gene ontology: tool for the unification of biology. The Gene Ontology Consortium, Nat. Genet. 25 (1) (2000) 25–29. 10802651 10.1038/75556 PMC3037419

[39] Kanehisa M, Goto S, KEGG: Kyoto encyclopedia of genes and genomes, Nucleic Acids Res. 28 (1) (2000) 27–30. 10592173 10.1093/nar/28.1.27 PMC102409

[40] Consortium TGO, Aleksander SA, Balhoff J, Carbon S, Cherry JM, Drabkin HJ, Ebert D, Feuermann M, Gaudet P, Harris NL, , The gene ontology knowledgebase in 2023, Genetics 224 (1) (2023). 10.1093/genetics/iyad031 PMC10158837 36866529

[41] Wang J, Vasaikar S, Shi Z, Greer M, Zhang B, WebGestalt 2017: a more comprehensive, powerful, flexible and interactive gene set enrichment analysis toolkit, Nucleic Acids Res. 45 (W1) (2017) W130–W137. 28472511 10.1093/nar/gkx356 PMC5570149

[42] Bodenhofer U, Kothmeier A, Hochreiter S, APCluster: an R package for affinity propagation clustering, Bioinformatics 27 (17) (2011) 2463–2464. 21737437 10.1093/bioinformatics/btr406

[43] Kim JE, Jeon S, Lindahl PA, Discovery of an unusual copper homeostatic mechanism in yeast cells respiring on minimal medium and an unexpectedly diverse labile copper pool, J. Biol. Chem. 299 (12) (2023) 105435. 37944620 10.1016/j.jbc.2023.105435 PMC10704325

[44] Peccarelli M, Scott TD, Wong H, Wang X, Kebaara BW, Regulation of *CTR2* mRNA by the nonsense-mediated mRNA decay pathway, Biochim. Biophys. Acta 1839 (11) (2014) 1283–1294. 25257758 10.1016/j.bbagrm.2014.09.011

[45] Eide DJ, Homeostatic and adaptive responses to zinc deficiency in Saccharomyces cerevisiae, J. Biol. Chem. 284 (28) (2009) 18565–18569. 19363031 10.1074/jbc.R900014200 PMC2707215

[46] Celik A, Baker R, He F, Jacobson A, High-resolution profiling of NMD targets in yeast reveals translational fidelity as a basis for substrate selection, RNA 23 (5) (2017) 735–748. 28209632 10.1261/rna.060541.116 PMC5393182

[47] Malabat C, Feuerbach F, Ma L, Saveanu C, Jacquier A, Quality control of transcription start site selection by nonsense-mediated-mRNA decay, Elife (2015) 4. 10.7554/eLife.06722 PMC4434318 25905671

[48] Singh G, Rebbapragada I, Lykke-Andersen J, A competition between stimulators and antagonists of Upf complex recruitment governs human nonsense-mediated mRNA decay, PLoS Biol. 6 (4) (2008) e111. 18447585 10.1371/journal.pbio.0060111 PMC2689706

[49] Poornima G, Srivastava G, Roy B, Kuttanda IA, Kurbah I, Rajyaguru PI, RGG-motif containing mRNA export factor Gbp2 acts as a translation repressor, RNA Biol. 18 (12) (2021) 2342–2353. 33910495 10.1080/15476286.2021.1910403 PMC8632111

[50] Windgassen M, Krebber H, Identification of Gbp2 as a novel poly(a)+ RNA-binding protein involved in the cytoplasmic delivery of messenger RNAs in yeast, EMBO Rep. 4 (3) (2003) 278–283. 12634846 10.1038/sj.embor.embor763 PMC1315891

[51] Miner GE, Sullivan KD, Zhang C, Hurst LR, Starr ML, Rivera-Kohr DA, Jones BC, Guo A, Fratti RA, Copper blocks V-ATPase activity and SNARE complex formation to inhibit yeast vacuole fusion, Traffic 20 (11) (2019) 841–850. 31368617 10.1111/tra.12683 PMC6800785

[52] Umebayashi K, Hirata A, Fukuda R, Horiuchi H, Ohta A, Takagi M, Accumulation of misfolded protein aggregates leads to the formation of Russell body-like dilated endoplasmic reticulum in yeast, Yeast 13 (11) (1997) 1009–1020. 9290205 10.1002/(SICI)1097-0061(19970915)13:11<1009::AID-YEA157>3.0.CO;2-K

[53] Oe S, Miyagawa K, Honma Y, Harada M, Copper induces hepatocyte injury due to the endoplasmic reticulum stress in cultured cells and patients with Wilson disease, Exp. Cell Res. 347 (1) (2016) 192–200. 27502587 10.1016/j.yexcr.2016.08.003

[54] Rees EM, Thiele DJ, Identification of a vacuole-associated metalloreductase and its role in Ctr2-mediated intracellular copper mobilization, J. Biol. Chem. 282 (30) (2007) 21629–21638. 17553781 10.1074/jbc.M703397200

[55] Linder MC, The relationship of copper to DNA damage and damage prevention in humans, Mutat. Res. 733 (1–2) (2012) 83–91. 23463874 10.1016/j.mrfmmm.2012.03.010

[56] Janke R, Kong J, Braberg H, Cantin G, Yates JR 3rd, Krogan NJ, Heyer WD, Nonsense-mediated decay regulates key components of homologous recombination, Nucleic Acids Res. 44 (11) (2016) 5218–5230. 27001511 10.1093/nar/gkw182 PMC4914092

[57] Dong J, Lai R, Nielsen K, Fekete CA, Qiu H, Hinnebusch AG, The essential ATP-binding cassette protein RLI1 functions in translation by promoting preinitiation complex assembly, J. Biol. Chem. 279 (40) (2004) 42157–42168. 15277527 10.1074/jbc.M404502200

[58] Young DJ, Guydosh NR, Zhang F, Hinnebusch AG, Green R, Rli1/ABCE1 recycles terminating ribosomes and controls translation Reinitiation in 3’UTRs in vivo, Cell 162 (4) (2015) 872–884. 26276635 10.1016/j.cell.2015.07.041 PMC4556345

[59] Khoshnevis S, Gross T, Rotte C, Baierlein C, Ficner R, Krebber H, The iron-Sulphur protein RNase L inhibitor functions in translation termination, EMBO Rep. 11 (3) (2010) 214–219. 20062004 10.1038/embor.2009.272 PMC2838684

[60] Zhu X, Zhang H, Mendell JT, Ribosome recycling by ABCE1 links lysosomal function and Iron homeostasis to 3’ UTR-directed regulation and nonsense-mediated decay, Cell Rep. 32 (2) (2020) 107895. 32668236 10.1016/j.celrep.2020.107895 PMC7433747

[61] Dancis A, Genetic analysis of iron uptake in the yeast Saccharomyces cerevisiae, J. Pediatr. 132 (3, Supplement) (1998) S24–S29. 9546033 10.1016/s0022-3476(98)70524-4

[62] Dancis A, Klausner RD, Hinnebusch AG, Barriocanal JG, Genetic evidence that ferric reductase is required for iron uptake in Saccharomyces cerevisiae, Mol. Cell. Biol. 10 (5) (1990) 2294–2301. 2183029 10.1128/mcb.10.5.2294-2301.1990 PMC360576

