# Supplementary material for: Global effect of copper excess and deficiency in Saccharomyces cerevisiae proficient or deficient in nonsense-mediated mRNA decay
Source: Genomics. Author manuscript; Available in PMC 2026 Jun 25. (PMC13295131; doi:10.1016/j.ygeno.2025.111020)
Supplement: 1 [file NIHMS2172113-supplement-1.pdf]

A

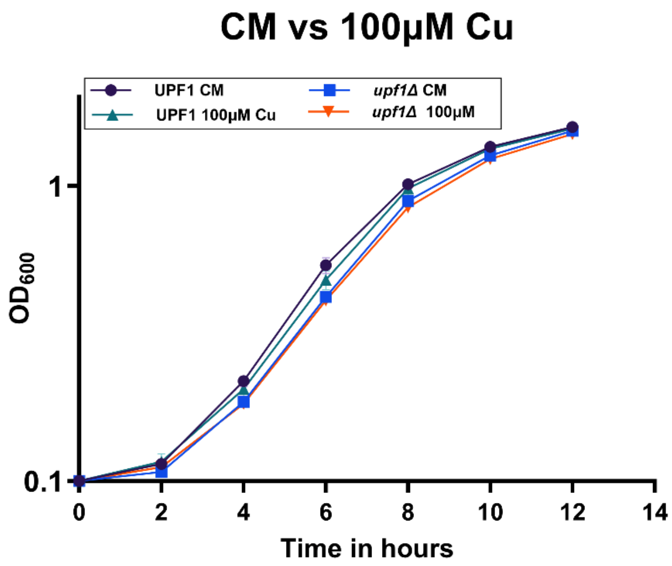

B

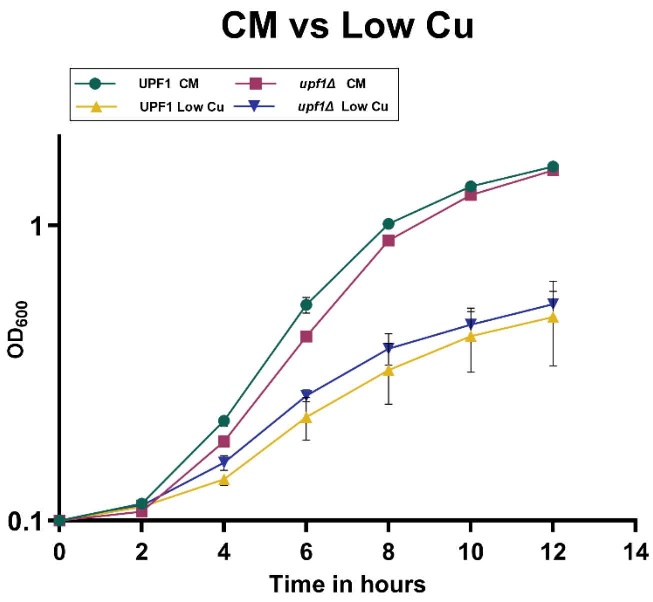

Supplementary Figure 1. Growth curves of wild-type and NMD mutant strains in complete minimal media, 100 μM copper (**A**) and low copper conditions (**B**).

A

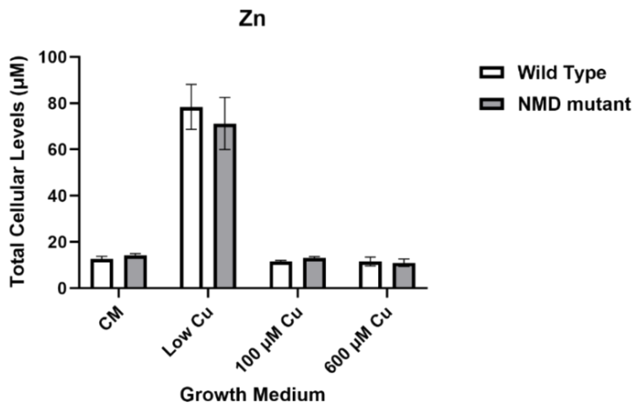

B

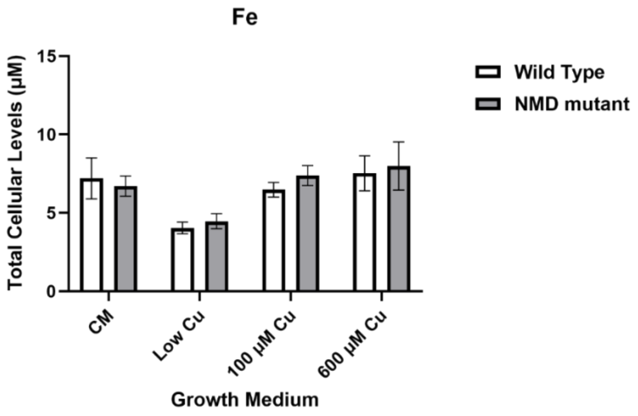

C

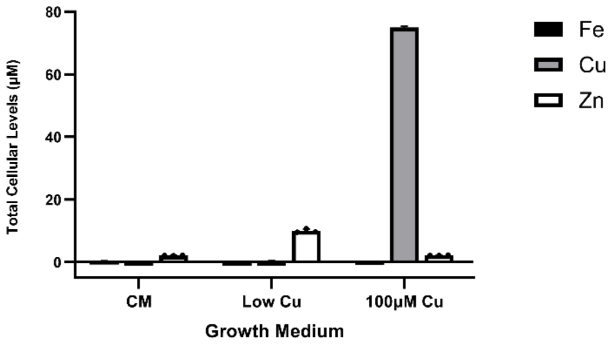

Supplementary Figure 2. Total amounts of Zinc (A) and Iron (B) levels in wild-type and NMD mutants grown in CM and low copper, 100 µM copper and 600 µM copper. Amounts of iron, copper and zinc in CM, Low copper and 100µM copper.

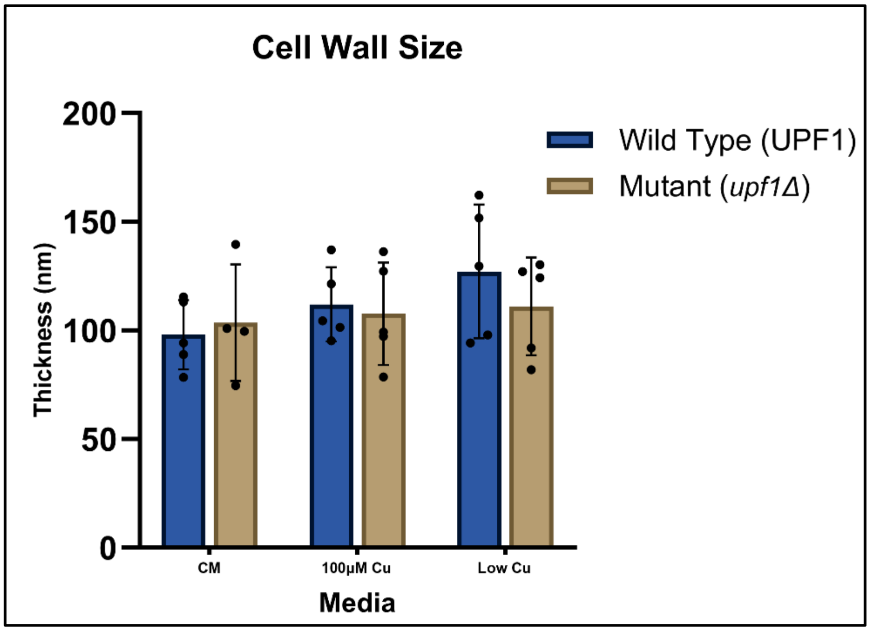

Supplementary Figure 3. Representative measurement of cell wall thickness (in nm) in CM, 100µM copper and low copper conditions.

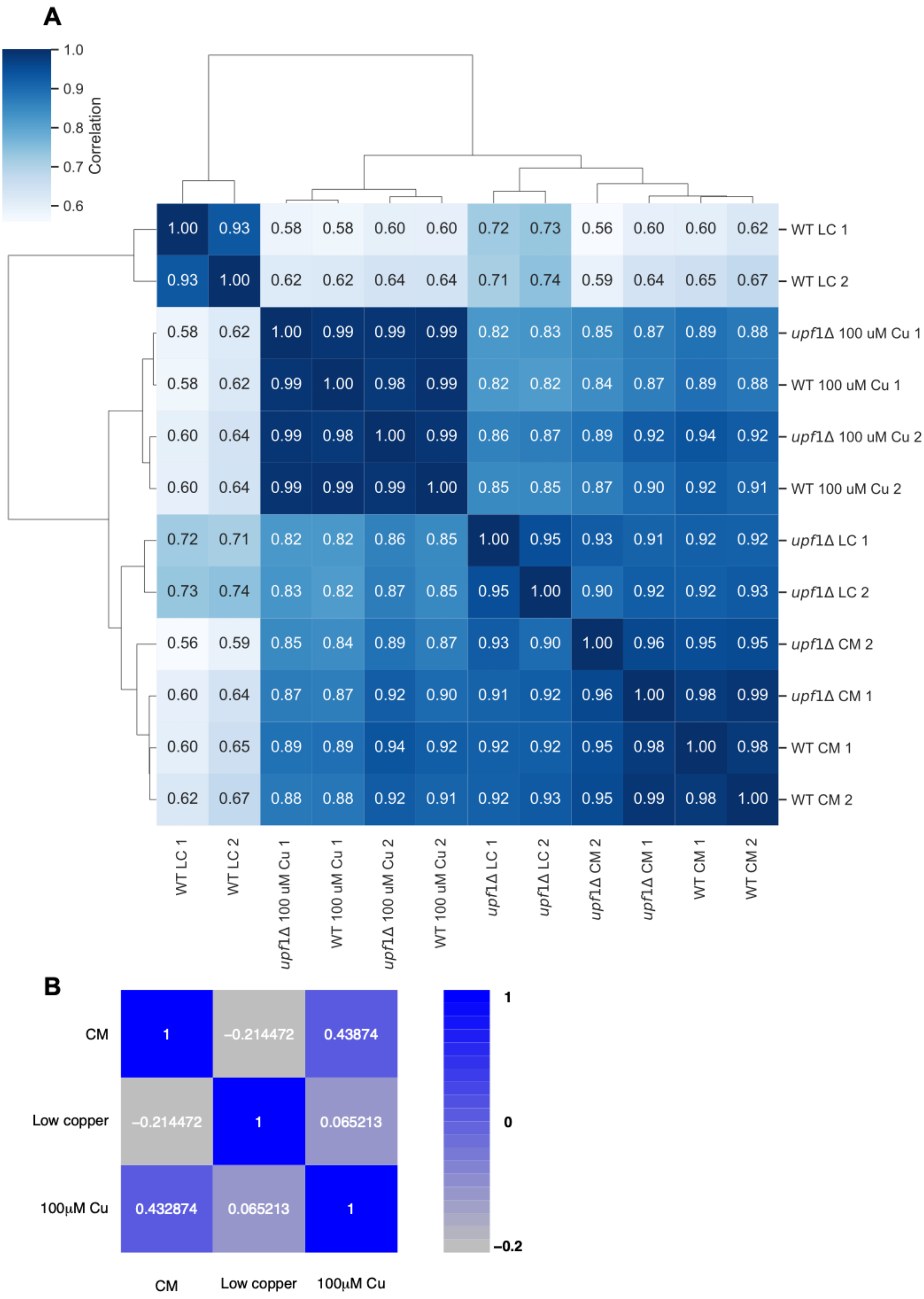
A
Correlation
1.0
0.9
0.8
0.7
0.6
1.00 0.93 0.58 0.58 0.60 0.60 0.72 0.73 0.56 0.60 0.60 0.62 WT LC 1
0.93 1.00 0.62 0.62 0.64 0.64 0.71 0.74 0.59 0.64 0.65 0.67 WT LC 2
0.58 0.62 1.00 0.99 0.99 0.99 0.82 0.83 0.85 0.87 0.89 0.88 upf1Δ 100 uM Cu 1
0.58 0.62 0.99 1.00 0.98 0.99 0.82 0.82 0.84 0.87 0.89 0.88 WT 100 uM Cu 1
0.60 0.64 0.99 0.98 1.00 0.99 0.86 0.87 0.89 0.92 0.94 0.92 upf1Δ 100 uM Cu 2
0.60 0.64 0.99 0.99 0.99 1.00 0.85 0.85 0.87 0.90 0.92 0.91 WT 100 uM Cu 2
0.72 0.71 0.82 0.82 0.86 0.85 1.00 0.95 0.93 0.91 0.92 0.92 upf1Δ LC 1
0.73 0.74 0.83 0.82 0.87 0.85 0.95 1.00 0.90 0.92 0.92 0.93 upf1Δ LC 2
0.56 0.59 0.85 0.84 0.89 0.87 0.93 0.90 1.00 0.96 0.95 0.95 upf1Δ CM 2
0.60 0.64 0.87 0.87 0.92 0.90 0.91 0.92 0.96 1.00 0.98 0.99 upf1Δ CM 1
0.60 0.65 0.89 0.89 0.94 0.92 0.92 0.92 0.95 0.98 1.00 0.98 WT CM 1
0.62 0.67 0.88 0.88 0.92 0.91 0.92 0.93 0.95 0.99 0.98 1.00 WT CM 2
WT LC 1
WT LC 2
upf1Δ 100 uM Cu 1
WT 100 uM Cu 1
upf1Δ 100 uM Cu 2
WT 100 uM Cu 2
upf1Δ LC 1
upf1Δ LC 2
upf1Δ CM 2
upf1Δ CM 1
WT CM 1
WT CM 2
B
CM 1 −0.214472 0.43874
Low copper −0.214472 1 0.065213
100μM Cu 0.432874 0.065213 1
1
0
−0.2
CM
Low copper
100μM Cu

Supplementary Figure 4. Correlation coefficient analysis between total RNA samples from wild-type (WT) and NMD mutant (*upf1Δ*) *S. cerevisiae* grown in CM, low copper (LC) and 100 μM copper (A) and between growth conditions (B).

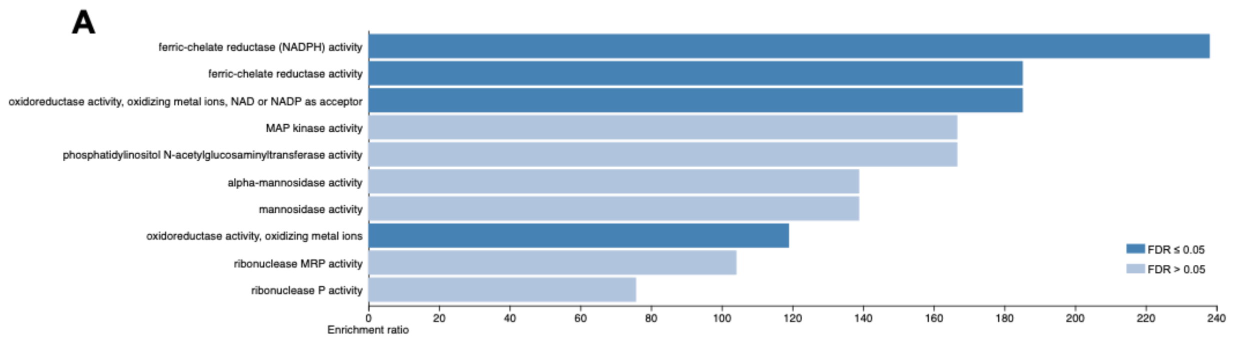

Supplementary Figure 5. Gene Ontology (GO) enrichments for Molecular Function terms for genes upregulated in low copper (LC) and 100 μM Cu (A).
